# An evolutionary medicine perspective on the design of non-hormonal contraceptives for women

**DOI:** 10.1093/emph/eoag008

**Published:** 2026-04-29

**Authors:** Ned J Place, Wei Yan

**Affiliations:** Department of Population Medicine & Diagnostic Sciences, College of Veterinary Medicine, Cornell University, S1-088 Schurman Hall, 240 Farrier Rd., Ithaca, NY 14853, USA; School of Molecular Biosciences and Center for Reproductive Biology, Washington State University, 1770 NE Stadium Way, Pullman, WA 99164, USA

**Keywords:** female contraceptives, evolutionary mismatches, reproductive life histories, unopposed oestrogen effects

## Abstract

Evolutionary mismatches between the reproductive life histories of women in industrialized societies and their Paleolithic ancestors are established risk factors for gynecological pathologies and breast cancer. In contemporary women, these include earlier menarche, delayed first full-term pregnancy, and reduced or absent lactation. Hormonal contraceptives lower the risks of ovarian and endometrial cancers by suppressing ovulation and modulating menstruation, and next-generation female contraceptives, including non-hormonal approaches, should aim to retain these non-contraceptive benefits. With careful design, non-hormonal contraceptives could further improve outcomes by reducing breast cancer risk, which may be variably elevated with oral contraceptive use. Importantly, these principles also have implications for male contraception, where preserving endocrine balance while achieving effective fertility control remains a central challenge. Evolutionary medicine provides a useful framework for developing contraceptives that optimize both reproductive and long-term health outcomes.

## INTRODUCTION

Half a century ago, Roger Short opined that ‘*Since natural selection has always operated in the past to maximize reproductive potential, women are physiologically ill-adapted to spend the greater part of their reproductive lives in the non-pregnant* [and non-lactating] *state*[s]’ [1].

Building on this insight, the risks of benign and malignant gynecological pathologies and breast cancer are heightened by the reproductive patterns that have become increasingly prevalent in industrialized societies and are markedly different from those of our evolutionary ancestors [2]. These risks are based on observations of contemporary hunter-gatherer societies, whose reproductive biology and behaviors can serve as a proxy for those of Paleolithic females, who were thought to have three-fold fewer lifetime ovulations and menstruations compared to modern-day women residing in industrialized countries owing to our ancestors’ later age of menarche (16 y), younger age at first birth (19.5 y), larger completed family size (6 children), and longer durations of lactation (3–4 y) [3, 4]. Additionally, the observations of Dogon women, who are contemporary millet farmers in Mali, West Africa, indicate we needn’t go all the way back to the Paleolithic to estimate the number of lifetime menstruations in women who practice natural fertility [5]. The median number of estimated lifetime menstruations, based on observations of menstrual hut visits was 109. The estimated median age of menarche was > 16 y, post-partum amenorrhea secondary to lactation was 20 months, and the total fertility rate was 8.6 ± live births per woman [6]. Many present-day women residing in modern societies could experience upwards of 400 lifetime ovulations and menstruations, representing evolutionary mismatches that contribute to the contemporary risks of benign and malignant gynecological pathologies and breast cancers [3, 4, 7].

Against this background, the risks of ovarian and endometrial cancers are generally thought to be reduced in women who have used combination oral contraceptive pills (OCPs) or long-acting, progestin-based contraceptives [8, 9]. However, these forms of hormonal contraceptives have the potential to variably increase the risk of breast cancer depending on the specific progestin formulation of the contraceptive and the duration of use, particularly for current and recent users [10–13]. Consequently, a large-scale transition from hormonal to non-hormonal contraceptives (NHCs) could negate reductions in the risks of ovarian and endometrial cancers that are associated with combination OCPs and progestin-only contraceptives. And independent of contraceptive use, the risks of gynecological and breast cancers are already relatively high as compared to our evolutionary ancestors owing to the younger age at menarche, later age at first full-term pregnancy, absent or shorter duration of lactation, hundreds of ovulatory and menstrual cycles, and the prevalence of obesity in industrialized societies.

For decades, having monthly menstruations for the vast majority of the reproductive lifespan has been considered normal [14]. Women are advised to consult with a medical professional if their menstrual patterns substantially deviate from the monthly cycle, which is entirely appropriate. However, from an evolutionary medicine perspective, experiencing upwards of 400 lifetime ovulations and menstruations is not ‘normal’ [4]. Despite this, there has been increasing emphasis on developing NHCs with mechanisms of action that would leave the hypothalamic–pituitary–gonadal (HPG) axis ‘undisturbed’, which would result in monthly ovulations and menstruations for the duration of usage. For example, the webpage for the Contraception Research Branch of the National Institute of Child Health and Human Development (NICHD) had previously posted that one of its research priorities was to support the development of ‘non-hormonal methods that do not disturb the hypothalamic-pituitary-gonadal axis’. While NHCs that leave the hypothalamic–pituitary–testicular (HPT) axis undisturbed might be an excellent idea (as discussed later), an undisturbed hypothalamic–pituitary–ovarian (HPO) axis over decades perpetuates an excessive number of lifetime ovulations and menstruations and the risks associated with them.

It is also important to recognize that female contraceptives currently in use, specifically hormonal contraceptives containing a progestin, are widely used to treat benign gynecological pathologies such as endometriosis, painful menses (dysmenorrhea), irregular menstrual cycles, and endometrial hyperplasia, and to reduce the risks of certain gynecological malignancies, including ovarian and endometrial cancers [8, 9, 15]. At the same time, these hormonal (steroidal) contraceptives are contraindicated and/or poorly tolerated by a substantial proportion of women, providing a strong impetus to develop new and improved female contraceptives, including NHCs.

Progestin-based hormonal contraceptives intended for continuous administration include combination OCPs, subcutaneous implants, long-acting (depo) intra-muscular injections, and progestin-releasing intra-uterine systems. Although originally developed solely to provide safe, effective, and reversible contraception, they have fortuitously demonstrated multiple additional benefits [15]. Multi-purpose preventive technology (MPT) traditionally refers to products that prevent unintended pregnancies (contraceptives) while also reducing the transmission of HIV and other sexually transmitted infections [16, 17]. In this context, combination OCPs and long-acting, progestin-only contraceptives represent a different form of MPT, as they provide effective contraception while simultaneously treating or preventing benign and malignant gynecological pathologies [8–10, 15]. As the development of new female contraceptives advances, including NHCs, it is therefore important not to lose sight of these multi-purpose, non-contraceptive benefits, and, ideally, to expand upon them.

**Figure 1:**
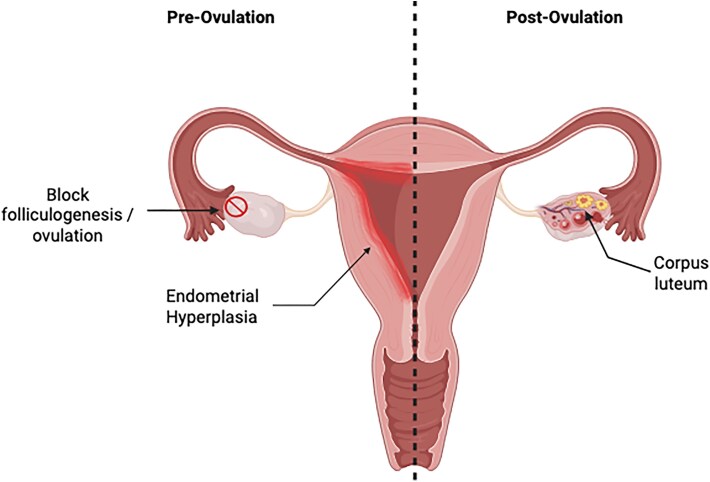
The mechanisms of action of non-hormonal contraceptives (NHCs) can be broadly categorized into those that block folliculogenesis and/or ovulation (pre-ovulatory) and those that block gamete transport and/or fertilization (post-ovulatory). Continuous use of NHCs with pre-ovulatory mechanisms of action would increase the risk of endometrial hyperplasia (left-side of figure) secondary to chronic anovulation and unopposed oestrogen effects (see also Fig. 2 and Explanatory Box 1). Created with BioRender.com.

For the purposes of this article, the objective is assumed to be the development of NHCs with pre-fertilization mechanisms of action, thereby avoiding their classification as abortifacients. Within this framework, NHCs can be broadly divided into those that block ovulation (pre-ovulatory) and those that block fertilization (post-ovulatory) (Fig. 1). Each category can be further subdivided by administration regimen, including continuous and on-demand use [18]. Importantly, NHCs that leave the HPO axis undisturbed must necessarily act post-ovulation, as any method that blocks ovulation inherently disrupts the HPO axis by preventing the formation of the corpus luteum (CL).

### Continuous use NHC with pre-ovulatory mechanism of action

**Figure 2:**
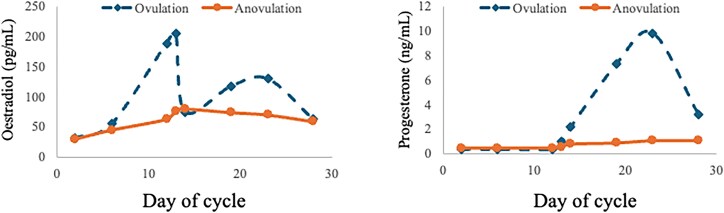
Oestradiol (E2) and progesterone (P4) concentrations during ovulatory and anovulatory cycles in women. Whereas the overall E2 concentrations during anovulatory cycles are lower than during ovulatory cycles, they are sufficient to have unopposed oestrogen effects on the endometrium when the luteal-phase P4 concentrations are ~ 30-fold lower in the absence of ovulation. This hormonal milieu will persist during the continuous use of non-hormonal contraceptives with a pre-ovulatory mechanism of action. With increasing duration of usage, hyperplastic changes in the endometrium (see Fig. 1) secondary to chronic anovulation results in the proliferative (mitotic) effects of oestrogen when it is unopposed by the differentiating and stabilizing effects of P4 concentrations that are produced by the corpus luteum during the luteal phase. Graphs in this figure were generated using data from Hambridge et al. [23], which is the work of the US government and not subject to domestic copyright protection under US law and is therefore in the public domain.

All components of the HPO axis are potential targets for NHCs that block ovulation. However, complete suppression of the entire axis, for example, via the administration of gonadotropin-releasing hormone (GnRH) antagonists or long-acting agonists, is undesirable because this would lead to a medically induced menopausal state of hypoestrogenaemia and all of its sequelae. Combination OCPs and long-acting progestin-only contraceptives (implants, depo-injections) prevent ovulation through feedback effects on hypothalamic GnRH secretion patterns that in turn disrupt the luteinizing hormone (LH) surge that immediately precedes and triggers ovulation [19]. Therefore, continuously used progestin-based contraceptives prevent ovulation at a late stage of folliculogenesis—the process by which non-growing primordial follicles are recruited into the growing pool of follicles. Typically, one follicle per cycle achieves a size and state of maturation that renders it responsive to the mid-cycle LH surge, which leads to ovulation of the dominant follicle [20]. The entire process from primordial follicle recruitment to ovulation was long thought to take ~ 1 year in women [21]; however, evidence is mounting that some follicles might enter a state of arrest and take substantially longer to complete folliculogenesis [22]. The search for potential NHC targets to block ovulation without causing hypoestrogenaemia is currently underway [23], and avoiding hypoestrogenaemia means NHC disruption of folliculogenesis must occur relatively late in the process, similar to when hormonal contraceptives have their effects. All of the potential NHC targets that might reversibly block ovulation are beyond the scope of this article, but suffice it to say that these methods are meant to maintain fairly normal concentrations of oestrogens. Blocking ovulation also blocks a critical physiological step that immediately follows, and that is the formation of the CL, which is the ovarian structure derived from the somatic follicular cells that had surrounded and supported the ovulated egg. The CL is the principal source of the increased concentrations of progesterone (P4) during the luteal phase of the cycle that are needed to prepare and maintain the endometrium for embryonic implantation and development. The menstrual cycle is clinically divided into two phases, the pre-ovulatory follicular phase and the post-ovulatory luteal phase, and P4 concentrations in the luteal phase are ~ 30-fold higher than in the follicular phase (Fig. 2) [24]. Maintenance of physiological oestrogen concentrations in the absence of luteal-phase P4 concentrations results in a phenomenon known as ‘unopposed oestrogen effects’ [25], which are described in Explanatory Box 1. Although precise population-level estimates vary by etiology and duration, the increased risk of endometrial hyperplasia and carcinoma associated with chronic anovulation and unopposed oestrogen is well established clinically, as evidenced by decades of data from women with polycystic ovary syndrome [28] and from uterus-intact post-menopausal women receiving unopposed oestrogen therapy [29]. The continuous use of an ovulation-blocking NHC and the resulting chronic anovulation would substantially increase the risks of endometrial hyperplasia, atypical hyperplasia (pre-cancerous), and cancer (Fig. 1) [26]. A potential solution to this problem is to combine an ovulation-blocking NHC with the minimal effective dose and duration of P4 supplementation to negate the risks of chronic anovulation. For example, micronized P4 at a replacement dosage is likely to be better tolerated than the synthetic progestins in hormonal contraceptives. However, this hybrid regimen could present logistical and compliance challenges if the continuous NHC and cyclical P4 administration cannot be administered together in a manner similar to combined OCPs, though the routes of NHC and supplemental P4 might be entirely different.

Explanatory Box 1 Chronic anovulation, unopposed oestrogen effects, and luteal-phase progesterone. Non-hormonal contraceptives (NHCs) with a pre-ovulatory mechanism of action, i.e. they block ovulation, and are meant to be taken continuously, will produce a state of chronic anovulation. Recognizing that the use of hormonal contraceptives, such as combination OCPs, sometimes spans decades, it’s not unreasonable to expect some individuals might use an ovulation-blocking NHC for durations of similar lengths. Chronic oligo- and anovulation are known risk factors for the development of endometrial cancer, which can be preceded by endometrial hyperplasia with or without atypia [26]. These changes in the endometrium are referred to as *unopposed oestrogen effects*, which is a phrase that is most often used in the context of menopausal hormone therapy (MHT). Uterus-intact women who are candidates for oestrogen replacement therapy (ERT) are strongly advised to co-administer a progestogen, either cyclically or continuously, to guard against the effects of unopposed oestrogen, specifically endometrial cancer [27]. Unopposed oestrogen effects also apply to women with chronic anovulation during their reproductive years, but in a somewhat different fashion. Rather than having progesterone (P4) concentrations in the extremely low post-menopausal range, these younger women have modest concentrations of P4, (Fig. 2); however, these levels aren’t sufficiently high to protect the endometrium from the oestrogen concentrations that are still present in pre-menopausal females. Menstrual cycles are generally divided into two phases, the follicular and luteal phases, which occur pre- and post-ovulation, respectively. Failure to ovulate precludes the formation of a corpus luteum, which is the principal source of increased P4 during the luteal phase. It is the absence of the higher P4 concentrations during the luteal phase (Fig. 2A) that predisposes women with chronic anovulation to develop endometrial hyperplasia and cancer due to the chronic proliferative (mitotic) effects of oestrogen when it is unopposed by the differentiating and stabilizing effects of P4 from the corpus luteum (Fig. 1). The lower P4 concentrations during the follicular phase do not provide this same protection. This state of chronic anovulation is perhaps best known in the context of polycystic ovarian syndrome (PCOS), and women with PCOS have an increased risk of developing endometrial cancer [28]. However, this cancer risk also applies to non-PCOS women who are chronically anovulatory for reasons other than using a contraceptive that contains a progestin. Chronic anovulation also predisposes women to abnormal uterine bleeding, which can be irregular, unpredictable in onset, and at times quite heavy. To prevent these sequelae, an ovulation-blocking NHC that is meant to be taken continuously might need to be accompanied by a P4 replacement regimen, or perhaps a selective progesterone receptor modulator, of a duration and dose that protects against the effects of unopposed endogenous oestrogen.

Of course, an NHC + P4 regimen wouldn’t be purely non-hormonal, but perhaps a hybrid system that avoids exposure to the supraphysiological concentrations of progestins in hormonal contraceptives would be an acceptable compromise. Finding a regimen of P4 supplementation that concurrently reduces the risks of endometrial and breast cancers is a laudable goal. Alternatively, if a potential NHC could induce a pre-ovulatory follicle to luteinize without it having to ovulate and produce sufficient P4 concentrations that protect against unopposed oestrogen effects on the endometrium, then a purely NHC without the need for P4 supplementation might be a possibility. Whereas luteinization of unruptured follicles (LUFs) via pharmacological induction is an experimental concept that presents challenges for clinical implementation, it has been the subject of preclinical studies [30], and a better understanding of the physiology and pathophysiology of LUF syndrome could be informative in the development of contraceptives with pre-ovulatory mechanisms of action [31].

### On-demand NHC with pre-ovulatory mechanism of action

In contrast to continuous-use approaches, pre-ovulatory NHCs can also be administered on demand. The currently available hormonal contraceptives that prevent (or delay) ovulation are the emergency contraceptive pills, which were once referred to as ‘morning-after pills’ and contain high dosages of a synthetic progestin (levonorgestrel). Another emergency contraceptive that has a somewhat different hormonal mechanism of action is ulipristal acetate, which is a selective progesterone receptor modulator. The progesterone receptor (PGR) is a ligand-activated nuclear transcription factor and an important modulator of ovulation. Genomics approaches are being used to interrogate pathways that are upstream and downstream of the PGR to identify potential targets for drugs that will acutely block ovulation [32]. Other investigators have developed an *ex vivo* ovulation system for the discovery of novel ovarian pathways and potential targets for NHC candidates [33].

The risks associated with on-demand NHCs, in terms of benign and malignant gynecological pathologies and breast cancer, might depend on the frequency and duration of usage. If an on-demand ovulation-blocking NHC is used in a manner that results in many anovulatory cycles, then the risk of endometrial cancer might still be increased without realizing a reduction in ovarian cancer risk. For combination OCPs, the degree to which the risk of ovarian cancer is reduced depends on the number of years of continuous use, and for endometrial cancer, the greatest risk reduction was observed in long-term OCP users who had compounding risk factors such as smoking and obesity [8]. Conversely, if the majority of cycles between menarche and menopause are ovulatory in women who use a pre-ovulatory NHC on-demand, then the risks of benign and malignant gynecological pathologies and breast cancer are likely to be similar to women who use post-ovulatory NHCs that leave the HPO axis undisturbed.

### Continuous-use and on-demand NHCs with post-ovulatory mechanism of action

NHCs with post-ovulatory mechanisms of action do not block ovulation and therefore fulfill the objective of leaving the HPO axis undisturbed. However, both continuous-use and on-demand NHCs that do not disturb the HPO axis in this category would still result in an excess number of lifetime ovulations and menstruations compared to our evolutionary ancestors, particularly in women who have no or few full-term pregnancies and who have either never lactated or have lactated for a relatively short-duration(s). The risks of gynecological and breast cancers associated with these reproductive histories are increased further by other attributes that are increasingly common in modern industrialized societies, which include older age at first birth in parous women, obesity, and many hundreds of ovulations and menstruations over their lifetimes [5, 34].

Nevertheless, for women in whom hormonal contraceptives are contraindicated or poorly tolerated, access to effective NHCs that leave the HPO axis undisturbed would represent a welcome alternative to the risks associated with unplanned pregnancies. The need for NHC options is particularly relevant during the peri-menopausal years, when irregular and anovulatory cycles become more common but the need for contraception hasn’t yet fully abated. In fact, preventing unintended pregnancy at this life stage may be especially important, given the increased prevalence of co-morbidities and poor pregnancy outcomes in this age group.

### Clarifying the meaning of ‘HPG axis undisturbed’

Having addressed some of the potential unintended consequences of NHCs that would leave the HPO axis undisturbed, it is worth revisiting the earlier statement from the NICHD Contraception Research Branch indicating a priority to develop ‘non-hormonal methods that do not disturb the HPG axis’. Importantly, the HPG axis also encompasses the HPT axis, which, unlike the HPO axis, has not been substantially perturbed over the course of hominin evolutionary history. As such, an evolutionary mismatch wouldn’t arise if spermatogenesis could be reversibly suppressed without the use of hormones. Accordingly, male NHCs that leave the HPT axis undisturbed would preserve endogenous sex steroid concentrations and avoid many of the shortcomings and stigma associated with low testosterone. In this respect, the logic underpinning non-hormonal male contraception aligns well with evolutionary medicine principles.

### Conceptual parallel between female and male endocrine preservation

Just as maintenance of endogenous oestrogen–progesterone balance is essential for physiological integrity and long-term health in women, preservation of endogenous testosterone production is critical in men. In women, disrupting this balance, for example, by suppressing ovulation without compensatory P4, can adversely affect endometrial health, mood, and metabolic function. Similarly, in men, suppressing endogenous testosterone through hormonal male contraceptive strategies requires careful consideration, as exogenous replacement may not fully recapitulate the nuanced, tissue-specific, and temporally regulated actions of endogenous androgens [35].

Extending this principle to male NHC development, the specific stage of spermatogenesis targeted is likely to be a critical determinant of endocrine neutrality. For male NHCs that target the mitotic (spermatogonia) or meiotic (spermatocyte) phases of spermatogenesis, substantial germ cell depletion may be unavoidable. It is well established that continuous, large-scale depletion of male germ cells within the seminiferous epithelium frequently activates the HPT axis, leading to elevations in follicle-stimulating hormone and/or LH [36, 37]. Importantly, the long-term consequences of persistently elevated gonadotropins, both on reproductive tissues and on systemic organs, remain poorly understood. In contrast, interventions targeting the haploid phase of spermatogenesis (spermiogenesis) rarely elicit a compensatory HPT response, because upstream germ cell populations and Sertoli–Leydig cell signaling remain largely intact [38, 39]. From the perspectives of both evolutionary medicine and endocrine homeostasis, spermiogenesis therefore represents a particularly attractive target for the development of truly non-hormonal male contraceptives.

Taken together, these parallels underscore a shared principle in reproductive endocrinology: contraceptive strategies should not merely block reproductive capacity but should also safeguard broader endocrine homeostasis. Recognizing this conceptual symmetry may help guide the design of safer and more physiologically harmonious contraceptive methods for both sexes.

## CONCLUSION

The considerations outlined here suggest that the central challenge in developing next-generation female NHCs is not simply identifying effective pre- or post-fertilization targets but integrating contraceptive efficacy with long-term endocrine and tissue health. From an evolutionary medicine perspective, design principles should therefore prioritize, where appropriate, minimizing lifetime ovulatory burden and avoiding chronic unopposed oestrogen exposure, rather than insisting on complete endocrine neutrality as an absolute goal. In this context, the relevant question is not whether a contraceptive is strictly ‘non-hormonal’, but whether its net physiological effects align with reproductive patterns under which women’s biology evolved.

This framework also argues for greater openness to hybrid contraceptive strategies. For continuous-use NHCs that block ovulation, limited or targeted P4 support, designed to restore luteal-phase functions without replicating the supraphysiological exposures of conventional hormonal contraceptives, may represent a more biologically realistic and clinically responsible approach than strictly hormone-free designs. Similarly, prioritizing post-ovulatory or on-demand strategies that preserve ovulation, or inducing luteinization without follicular rupture, may offer alternative paths that balance contraceptive efficacy with reduced long-term risk. Rather than viewing hormonal exposure as inherently undesirable, future contraceptive development may benefit from treating hormones as precision tools to be used sparingly and strategically in service of broader health goals.

Ultimately, evolutionary mismatches between modern reproductive life histories and those of our Paleolithic ancestors continue to shape risks of benign and malignant gynecological pathologies and breast cancer. As efforts to develop new female contraceptives accelerate, maintaining and, where possible, improving the multi-purpose health benefits of existing methods should remain a core design objective. By applying evolutionary medicine as a guiding framework, contraceptive innovation can move beyond binary distinctions between hormonal and non-hormonal approaches toward solutions chosen, in Roger Short’s words [1], ‘with great care’, in recognition of their long-term consequences for women’s health and well-being.
